# Higher Circulating Trimethylamine N-Oxide Aggravates Cognitive Impairment Probably via Downregulating Hippocampal SIRT1 in Vascular Dementia Rats

**DOI:** 10.3390/cells11223650

**Published:** 2022-11-17

**Authors:** Yang Deng, Junqing Zou, Ye Hong, Qiang Peng, Xinxin Fu, Rui Duan, Jie Chen, Xiangliang Chen

**Affiliations:** 1 Department of Neurology, Nanjing First Hospital, China Pharmaceutical University, Nanjing 210006, China; 2 Department of Neurology, Nanjing First Hospital, Nanjing Medical University, Nanjing 210006, China; 3 Department of Personnel, Nanjing First Hospital, Nanjing 210006, China

**Keywords:** trimethylamine N-oxide, SIRT1, inflammation, oxidative stress, synaptic plasticity, vascular dementia

## Abstract

Oxidative stress and inflammation damage play pivotal roles in vascular dementia (VaD). Trimethylamine N-oxide (TMAO), an intestinal microbiota-stemming metabolite, was reported to promote inflammation and oxidative stress, involved in the etiology of several diseases. Still, these effects have not been investigated in VaD. Here, we tested whether pre-existing, circulating, high levels of TMAO could affect VaD-induced cognitive decline. TMAO (120 mg/kg) was given to rats for a total of 8 weeks, and these rats underwent a sham operation or bilateral common carotid artery (2VO) surgery after 4 weeks of treatment. Four weeks after surgery, the 2VO rats exhibited hippocampal-dependent cognitive function declines and synaptic plasticity dysfunction, accompanied by an increase in oxidative stress, neuroinflammation, and apoptosis. TMAO administration, which increased plasma and hippocampal TMAO at 4 weeks postoperatively, further aggravated these effects, resulting in exaggerated cognitive and synaptic plasticity impairment, though not within the Sham group. Moreover, TMAO treatment activated the NLRP3 inflammasome and decreased SIRT1 protein expression within the hippocampus. However, these effects of TMAO were significantly attenuated by the overexpression of SIRT1. Our findings suggest that TMAO increases oxidative stress-induced neuroinflammation and apoptosis by inhibiting the SIRT1 pathway, thereby exacerbating cognitive dysfunction and neuropathological changes in VaD rats.

## 1. Introduction

Vascular dementia (VaD) reflects the medical syndrome of having cognitive issues due to multiple cerebrovascular factors [1,2]. Such manifestations include memory losses when experiencing incidents, emotional disturbances, and misunderstandings [3]. Epidemiological findings suggest that approximately 35,600,000 individuals suffer from VaD, with this statistic expected to reach 100,000,000 prior to 2050 [4]. However, there is currently no effective clinically approved drug for treating VaD. Therefore, it is extremely imperative to probe pathological changes together with the possible molecular mechanisms of cognitive dysfunction within VaD.

Previous studies have shown that oxidative stress and neuroinflammation form the two main variables for VaD pathogenesis [5,6]. Excessive ROS are generated during oxidative stress, and the accumulation of ROS disrupts the balance across the oxidative and antioxidant systems, which can act as redox signaling molecules to activate or regulate NLRP3 inflammasome triggering [7]. Once activated, NLRP3 recruits ASC, promoting the conversion of pro-caspase-1 to cleaved caspase-1, subsequently driving pro-IL-1β cleaving into its mature forms [8]. As a class III histone deacetylase, silent information regulator 1 (SIRT1) is involved within multiple biological processes such as cell cycle regulation, oxidative stress, and inflammation responses [9,10]. SIRT1 was also implicated within regulating NLRP3 inflammasome triggering [11,12,13]. Furthermore, SIRT1 regulates BDNF expression and is considered a strong regulator of synaptic plasticity and cognitive function [14,15]. However, whether SIRT1 regulates NLRP3 inflammasome activation and improves cognitive capacity within VaD rats still requires clarification.

Growing evidence suggests that changes within intestinal microbiota remain strongly linked with human health and disease [14,15]. Trimethylamine N-oxide (TMAO), a specific dietary nutrient metabolite stemming from intestinal microbiota, has been proven to play pivotal parts within cardiovascular and central nervous system diseases [15,16,17]. Within the intestines, the intestinal microbiota converts ingested precursors such as L-carnitine into trimethylamine (TMA), subsequently transforming into TMAO through hepatic flavin monooxygenase 3 (FMO3) [18]. One particular investigation found TMAO concentrations to be markedly elevated within the circulation and cerebrospinal fluid (CSF) in Alzheimer’s disease (AD) and mild cognitive impairment cases [19]. Elevated TMAO triggers oxidative stress and NLRP3 inflammasome activation, leading to the increased production of inflammatory cytokines [20,21]. Furthermore, elevated TMAO has been revealed to be mechanistically relevant to cognitive impairment by aggravating synaptic damage [22]. Furthermore, Ke et al. observed that the elevation of circulating TMAO during aging may worsen endothelial and vascular function by inhibiting SIRT1 expression and increasing oxidative stress [23]. However, whether high levels of circulating TMAO levels aggravate VaD cognitive impairment by regulating SIRT1 remains uncertain.

This investigation employed a rat model for bilateral common carotid artery occlusion in order to probe TMAO’s influence on vascular dementia and to determine whether this effect was mediated via regulating the expression of SIRT1.

## 2. Materials and Methods

### 2.1. Animals

Male Sprague-Dawley rats (weight 280–300 g; 8 weeks old) were purchased from the Model Animal Research Center of Nanjing University (Nanjing, China). All rats were kept within an animal chamber with fixed heating (21 ± 2 °C), relative humidity (50 ± 5%), a 12-h dark/light cycle, and ad libitum feed. All assays were performed through compliance with National Institutes of Health directives and were accepted through the Animal Research and Ethics Committee of Nanjing First Hospital (protocol number: DWSY-2104386).

### 2.2. TMAO Treatment and Experimental Model

The animals were segregated in a randomized manner within six groups: Sham operation group (Sham); TMAO group (TMAO); 2-vessel occlusion group (2VO); 2VO + TMAO group (2VO + TMAO); 2VO + TMAO + LV-NC group (2VO + TMAO + LV-NC); and 2VO + TMAO + LV-SIRT1 group (2VO + TMAO + LV-SIRT1). Rats were given water solubilized with TMAO (120 mg/kg) for 4 consecutive weeks before 2VO modeling [24], whereas mice within the Sham group were fed with sterile water. The rats were anesthetized with 4% isoflurane at 48 h before surgery and positioned on a stereotaxic device (RWD Life Science, San Diego, CA, USA). The 5 μL of lentivirus (negative lentivirus and LV-SIRT1; Gene Pharma Company, Shanghai, China) was directly injected into the right ventricles of the rats [25]. Thereafter, 2VO modeling was performed to generate a global cerebral hypoperfusion model according to previous studies [26,27]. In brief, a 2-cm middle incision exposed the bilateral common carotid arteries, which were gently separated from the cervical sympathetic and vagus nerves, followed by being ligated in a permanent manner through a 4–0 silk suture. The Sham operation group involved performing the same surgery but excluding the ligating step. TMAO was continuously administered for 4 weeks from the second day after the operation, followed by behavioral testing. Animals were subsequently euthanized so that blood and brain hippocampus samples could be collected for biochemical and molecular purposes.

### 2.3. Determination of TMAO in Plasma and Hippocampal Samples via High-Performance Liquid Chromatography–Tandem Mass Spectrometry (HPLC–MS/MS)

TMAO plasma and hippocampal concentrations were detected through HPLC–MS/MS. Briefly, 60 μL of samples were aliquoted into 1.5-mL Axygen tubes in combination with 10 μL of a 1 ug/mL internal standard consisting of d9-TMAO within methanol. Sample proteins were centrifuge-extracted, while the supernatants (5 μL) were analyzed by injection into a Waters BEH C18 column (2.1 × 50 mm, 1.7 μm, cat. no. 03433919615148; Milford, MA, USA), running at a flow rate of 0.4 mL/min with an LC-20AD Shimadzu pump system and SIL-20AXR autosampler connected to a Triple Quad 4500MD mass spectrometer (AB SCIEX, Framingham, MA, USA). By mixing solvent A (0.1% formic acid within water) and solvent B (0.1% formic acid within methanol) at varying ratios, a discontinuous gradient was generated, starting with 10% B for analyte separation, then increasing in a linear manner up to 80% B over 1.0 min, maintained for 1.8 min, with a consequent return to 10% B. The TMAO was quantified by using multiple reaction monitoring (MRM) transitions at m/z 76.1→59, and the d9-TMAO, at m/z 85.1→68.1

### 2.4. Open Field Test (OFT)

The general locomotor activity of the rats was assessed by OFT, previously described in [28]. The rats were positioned in the center of a black box (100 × 100 × 40 cm) and then moved freely. The time spent and the total travel distance into the central area were recorded with the camera linked to an Any-maze^®^ tracker (New Soft Information Technology Co., Ltd.™., Shanghai, China). The chamber was cleaned with 75% ethanol and dried after each trial to avoid olfactory cues.

### 2.5. Morris Water Maze Test (MWM)

An MWM test was conducted to evaluate the spatial learning and memory capacity of the rats [29]. Briefly, the MWM device was composed of a round, black, opaque water pool, divided equally into four quadrants. A transparent circular stage was positioned 2.00 cm beneath the water surface. An EthoVision 3.0 automated animal video tracker (Noldus Information Technology B.V.™, Wageningen, Netherlands) was employed. Four training trials were performed on each rat for 5 consecutive days. For each day, the rats were placed at random into the water from four points until they found the stage and stayed there for 15 s within 60 s. The rats were led to the submerged platform when they could not find it in less than 60 s. During the next day of the final trial, the path length of the rats to the merged stage was recorded after the removal of the stage, and the means of the four trials were determined.

### 2.6. Nissl Staining

Nissl staining was performed as mentioned in [30]. Briefly, paraffin-coated segments were deparaffinized and rehydrated, followed by tar violet treatment at 56 °C for 1 h. After that, the segments were desiccated with ethanol gradient concentrations, xylene-rinsed, and neutral gum-sealed once the dye removal was completed. Six fields of hippocampus were randomly selected on each coronal section using an Olympus microscope (Olympus™, Tokyo, Japan) for quantitative analysis. Nuclei with round cell bodies, cytoplasmic Nissl material, and visible nucleoli were identified as normal neurons, whereas nuclei with a disorganized arrangement, Nissl material disappearance, surrounded by voids, and unrecognizable nucleoli were identified as damaged neurons. Nissl-positive neurons % = positive neurons/total neurons × 100%.

### 2.7. Immunofluorescence

The rats were exposed to a cold PBS and 4% paraformaldehyde perfusion after anesthesia. The rat brains were removed, fixated, desiccated, and sliced into 10-μm segments. The segments were permeabilized, blocked, and followed by incubation with the primary antibodies anti-NLRP3 (1:100, #DF7438; Affinity Biosciences, Cincinnati, OH, USA), anti-SIRT1 (1:100, #8469; CST, Boston, MA, USA), anti-8-OHdG (1:100, sc-66036; Santa Cruz Biotechnology, CA, USA), anti-MAP2 (1:50, #4542; CST, Boston, MA, USA), and anti-NeuN (1:100, ab177487; Abcam, Cambridge, MA, USA), overnight at 4 °C. Following triple TBST wash steps, the segments were placed under incubation with the corresponding, fluorescent, secondary antibody. Subsequently, the segments were sealed with DAPI (C1006; Beyotime, Shanghai, China), imaged with a laser scanning confocal microscope (ZEISS 8.0, Oberko, Germany), and quantified by Image J software.

### 2.8. Immunohistochemistry (IHC)

The rat brain sections were deparaffinized, dehydrated, incubated, and then repaired with antigen. After cooling, the sections were permeabilized, blocked, and incubated with the primary antibodies NLRP3 (1:100, #DF7438; Affinity), anti-3-NT (1:100, ab61392; Abcam), and anti-SIRT1 (1:100, #8469; CST), overnight at 4 °C. Next, the sections were placed under incubation with the corresponding biotinylated secondary antibody. The sections were visualized with 3,3-diaminobenzidine (DAB) staining (Absin, Shanghai, China) and kept in a dark room for 8 min. All images were gained with an Olympus microscope (Olympus™, Tokyo, Japan) and quantitatively analyzed by Image J software.

### 2.9. Golgi Staining

Golgi staining revealed subtle morphological changes in the neuronal dendritic spines of the samples. The Golgi staining steps were conducted following the manufacturer’s instructions (#PK401A; D Neurotechnologies™, Columbia, USA) [31]. Briefly, the rat brain tissues were placed within a tube containing a mixture of solutions A and B, in a light-free environment for 2 weeks, and later incubated with solution C for 3 days. Coronal sections (100 μm) were cut with a cryostat (Leica, Wetzlar, Germany). The sections were then stained, dehydrated, and cleared with xylene, followed by neutral resin. Neuronal images were obtained with an Olympus microscope (Olympus™, Tokyo, Japan). The numbers of apical dendritic spines were quantified using Image J. Results are presented as the number of apical dendritic spines per 10 μm.

### 2.10. Oxidative Stress Parameters Measurement

The rat hippocampus tissue was well-homogenized with cold saline followed by 4 °C centrifuging. The supernatant was consequently collected and measured with the appropriate kits for malondialdehyde (MDA; cat. no. A003-1-2), protein carbonyl compound (PCC; cat. no. A087-1-2), superoxide dismutase (SOD; cat. no. A001-3-2), and glutathione peroxidase (GPX; cat. no. A005-1-1) content following the kit protocols, procured from the Nanjing Jiancheng Bioengineering Institute (Nanjing, China).

### 2.11. Terminal Deoxynucleotidyl Transferase dUTP Nick-End Labeling (TUNEL) Assay

TUNEL staining of the paraffin-embedded brain segments was conducted using an assay kit (Roche Inc., Indianapolis, IN, USA) following the kit protocols. The percentage of TUNEL-positive cells within the chosen microscopic field for the hippocampus was quantified by Image J software.

### 2.12. Western Blot Analysis

Rat hippocampal tissues were directly separated on ice, homogenized with ice-cold RIPA lysate buffer, and centrifuged at 4 °C. The supernatant was collected to quantify the protein concentration using a Pierce BCA Protein Assay Kit^®^ (Thermo Fisher Scientific, Inc.™, Carlsbad, CA, USA). The proteins were separated through different SDS-PAGE methods as well as transferred to PVDF membranes. The membranes were blocked and incubated with anti-PSD-95 (1:2000, cat. no. 20665-1-AP; Proteintech, Wuhan, China), anti-MAP-2 (1:1000, #4542; CST, Boston, MA, USA), anti-synaptophysin (1:1500, #SAB4502906; Sigma-Aldrich, St.Louis, MO, USA), anti-NLRP3 (1:1000, #DF7438; Affinity Biosciences, Cincinnati, OH, USA), anti-IL-1β (1:1000, ab254360; Abcam, Cambridge, MA, USA), anti-Bcl-2 (1:1000, ab196495; Abcam, Cambridge, MA, USA), anti-Bax (1:1000, #2772; CST, Boston, MA, USA), anti-CL-caspase-3 (1:1000, #9661; CST, Boston, MA, USA), anti-SIRT1 (1:1000, #8469; CST, Boston, MA, USA), or β-actin (1:1000, #4970; CST, Boston, MA, USA), overnight at 4 °C. The membranes were placed under incubation with horseradish peroxidase-conjugated secondary antibodies (1:3000; CST, Boston, MA, USA). Finally, the visualization of bands was performed using an ECL kit (Millipore™, Merck, Darmstadt, Germany) and the protein band density was quantified with Image J. All data are presented with β-actin as the standard.

### 2.13. Statistical Analysis

The data of all experiments were reflected as the mean ± standard deviation (SD) and analyzed through Prism 8 software (GraphPad™, San Diego, CA, USA). The escape latency in the MWM test was analyzed by a two-way repeated measures analysis of variance (ANOVA) for the variation across the groups. Other data were subjected to multiple comparisons by a one-way ANOVA and subsequent Tukey–Kramer post hoc analysis. *p* < 0.05 conferred statistical significance. A minimum of three runs was performed for all experiments.

## 3. Results

### 3.1. The Levels of TMAO Increased in TMAO-Treated Rats after 2VO Surgery

**Figure 1:**
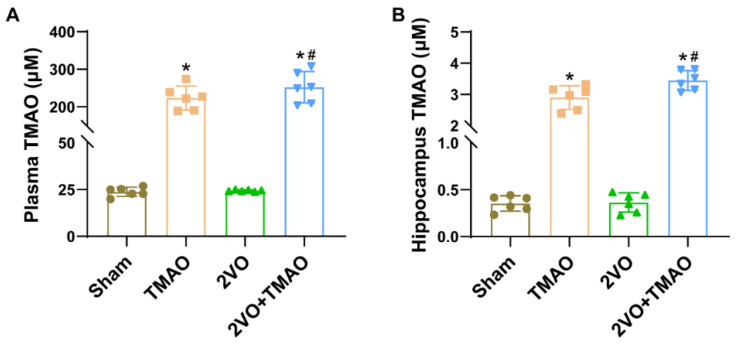
The levels of TMAO increased in TMAO-treated rats after 2VO surgery. TMAO levels in (**A**) plasma and (**B**) hippocampal tissue treated with TMAO before and 4 weeks after 2VO surgery (n = 6). The datasets reflect the mean ± SD. * p < 0.05 versus the Sham group; ^#^ *p* < 0.05 versus the 2VO group.

Changes in the TMAO levels in the plasma and hippocampi of the rats after TMAO treatment were measured via HPLC–MS/MS. It was found that the dosage of TMAO at 120 mg/kg administered in potable water continuously throughout 8 weeks, drove marked increases in the TMAO plasma levels across both the TMAO and 2VO+TMAO groups (Figure 1A). The TMAO content analysis of the hippocampal tissue revealed that treatment with TMAO also increased TMAO tissue content in both groups receiving TMAO (Figure 1B). Such dataset outcomes suggest TMAO solution administration markedly upregulated the TMAO plasma and hippocampal levels in the vascular dementia rats.

### 3.2. TMAO Aggravated Cognitive Function Deficits in 2VO Rats

**Figure 2:**
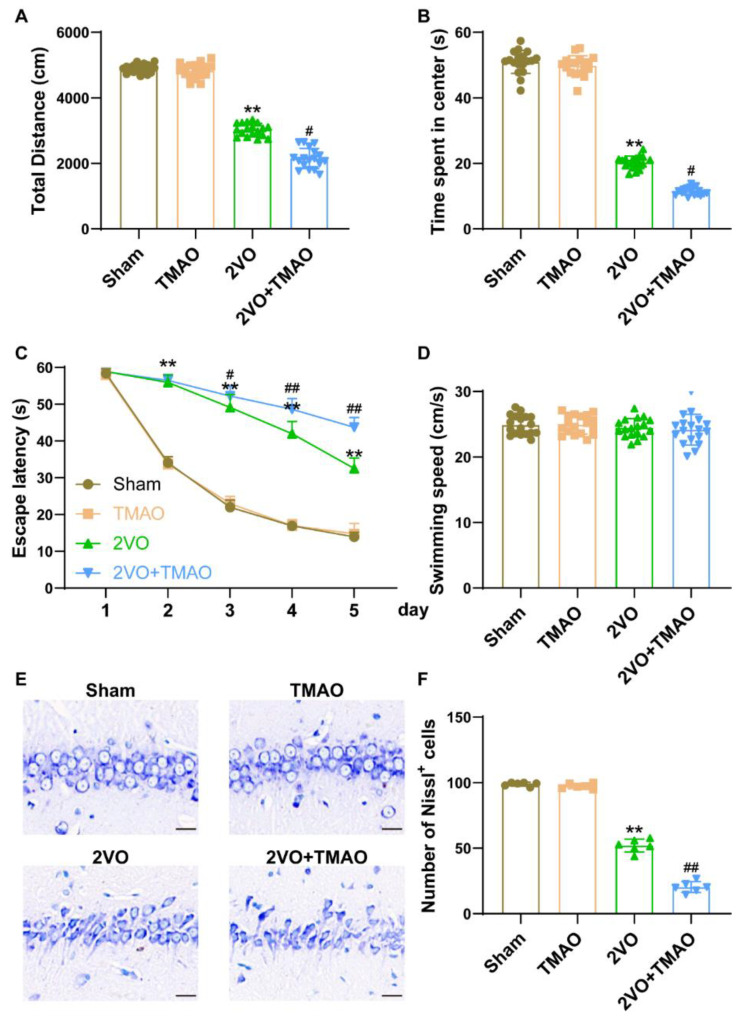
TMAO aggravated cognitive function deficits in 2VO rats. The (**A**) total distance traveled and (**B**) duration of time spent at the center across the groups were measured using an open field test (n = 18). The (**C**) escape latency and (**D**) swimming speeds across the groups were recorded during the hidden stage task of rats 4 weeks after 2VO surgery (n = 18). (**E**) Images reflecting neuronal injuries within the hippocampal CA1 area of the rats across the groups were detected via Nissl staining. Scale bar, 20 μm (n = 6). (**F**) Quantification of the Nissl^+^ cells is shown as a bar chart (n = 6). Datasets reflect the mean ± SD. ** *p* < 0.01 versus the Sham group; ^#^ *p* < 0.05 and ^##^ *p* < 0.01 versus the 2VO group.

For the purpose of determining the TMAO treatment influences on the learning and memory capacities of the 2VO rats, OFT and MWM assays were conducted. In comparison with the Sham rats during OFT, the total distance traveled and the percentage of time spent in the center were largely reduced within the 2VO rats (Figure 2A,B). The decline within these parameters was further exacerbated in TMAO-treated 2VO rats, suggesting that TMAO treatment attenuated the locomotor ability. Subsequently, MWM dataset outcomes suggest that the mean escape latency was extended within the 2VO rats, unlike in the Sham group, with TMAO administration prolonging such extended escape latency (Figure 2C). No variation was observed across the swimming speeds for all groups (Figure 2D). These findings demonstrate that TMAO could aggravate the cognitive impairment triggered by 2VO. Furthermore, Nissl staining assessed TMAO’s influence on hippocampal neuronal injury within the 2VO operated rats. Unlike the Sham group, Nissl-positive cells in the CA1 area of the 2VO rat hippocampus samples were reduced. TMAO treatment markedly increased neuronal loss within the CA1 area, induced by 2VO (Figure 2E,F).

### 3.3. TMAO Attenuated Hippocampal Synaptic Plasticity within 2VO Rats

**Figure 3:**
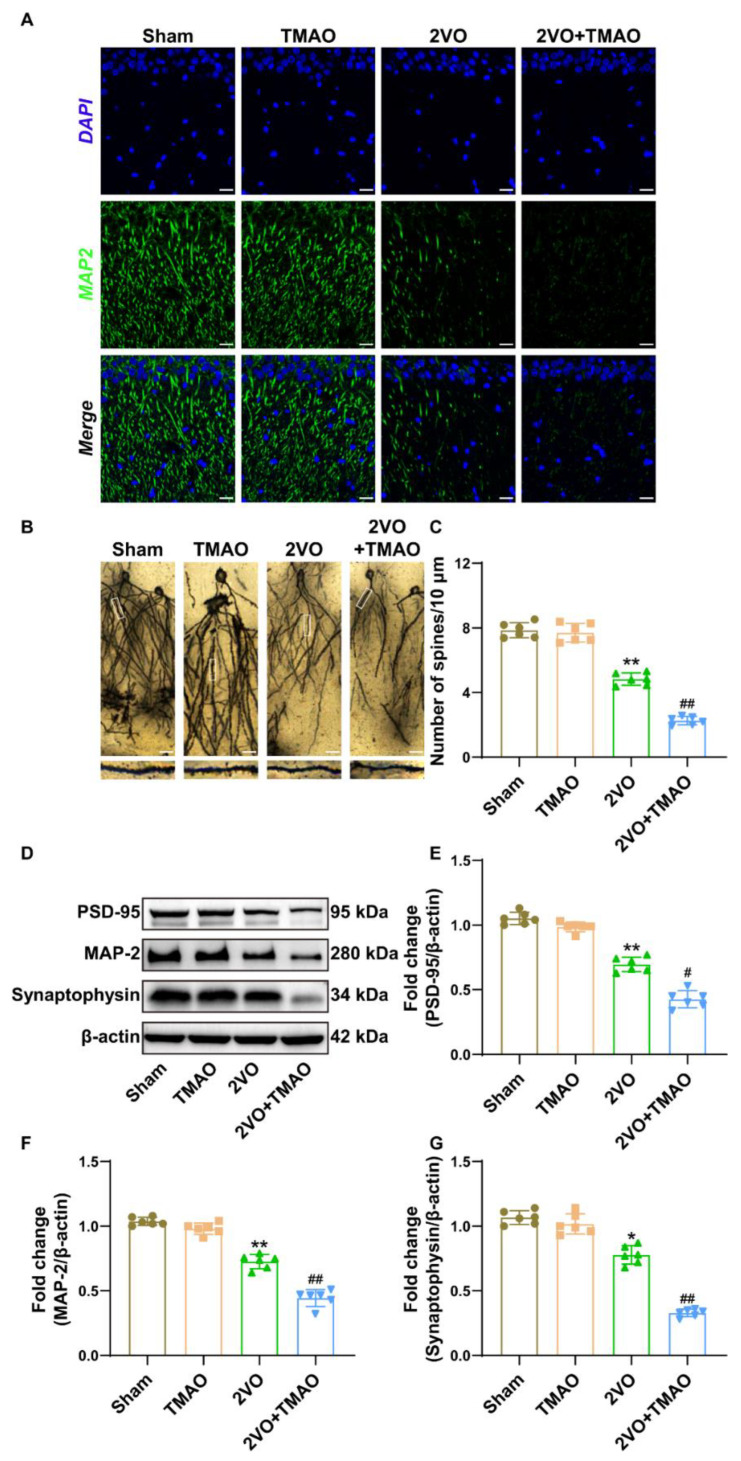
TMAO attenuated hippocampal synaptic plasticity within 2VO rats. (**A**) Representative immunostaining showing the neuronal structures stained with MAP-2 (green) within the hippocampal CA1 area. Nuclei were subjected to DAPI staining. Scale bar, 20 μm (n = 6). (**B**) Representative imaging for Golgi-stained neuronal structures within the hippocampus. Scale bar, 20 μm. Micrographs are shown at 200× (n = 6). (**C**) Quantification of apical dendritic spine densities is shown as a bar chart (n = 6). (**D**) Representative bands of PSD95, MAP-2, and synaptophysin protein expressions within the rat hippocampus across the groups. β-actin served as a loading control (n = 6). Quantitative assessment for (**E**) PSD95, (**F**) MAP-2, and (**G**) synaptophysin protein expression is shown as a bar chart (n = 6). Datasets reflect the mean ± SD. * *p* < 0.05 and ** *p* < 0.01 versus the Sham group; ^#^ *p* < 0.05 and ^##^ *p* < 0.01 versus the 2VO group.

Due to the presence of dendritic spine loss within the VaD rat cerebra [32], an investigation of the neuronal marker MAP-2 and apical dendritic spine densities within the hippocampal CA1 area was necessary. The immunofluorescence staining dataset outcomes indicate marked downregulation in MAP-2 within the hippocampal CA1 area for the 2VO rats exposed to TMAO (Figure 3A). The density of the apical dendritic spines was clearly damaged in the 2VO rats and TMAO treatment aggravated the damage of the apical dendritic spines (Figure 3B,C). Synaptic plasticity-related proteins including PSD95, MAP-2, and synaptophysin are a critical part of the brain learning mechanism [33]. Figure 3D–G depicts that the protein expression of PSD95, MAP-2, and synaptophysin was evidently reduced after 2VO treatment. TMAO treatment further downregulated these protein expressions. Altogether, these data suggest that TMAO downregulated synaptic plasticity-associated proteins together with impairing synapse function in the 2VO rats.

### 3.4. TMAO Induced Neuronal Apoptosis within 2VO Rats

**Figure 4:**
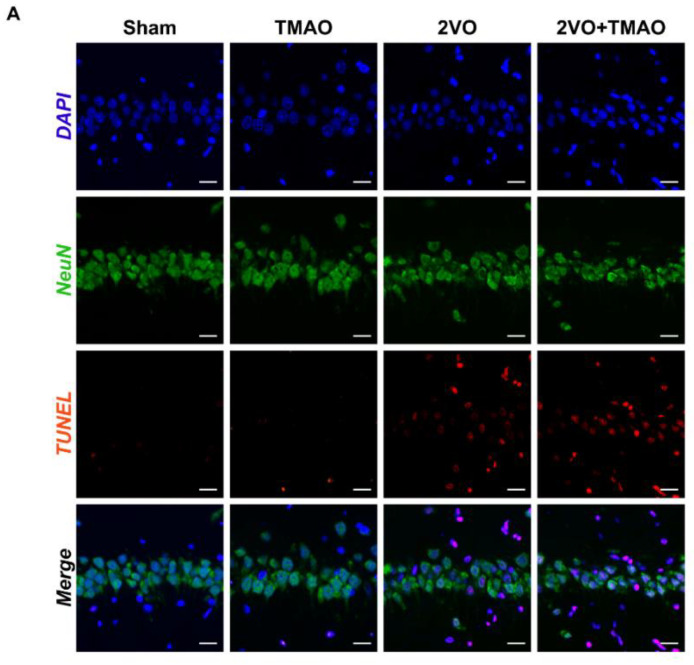

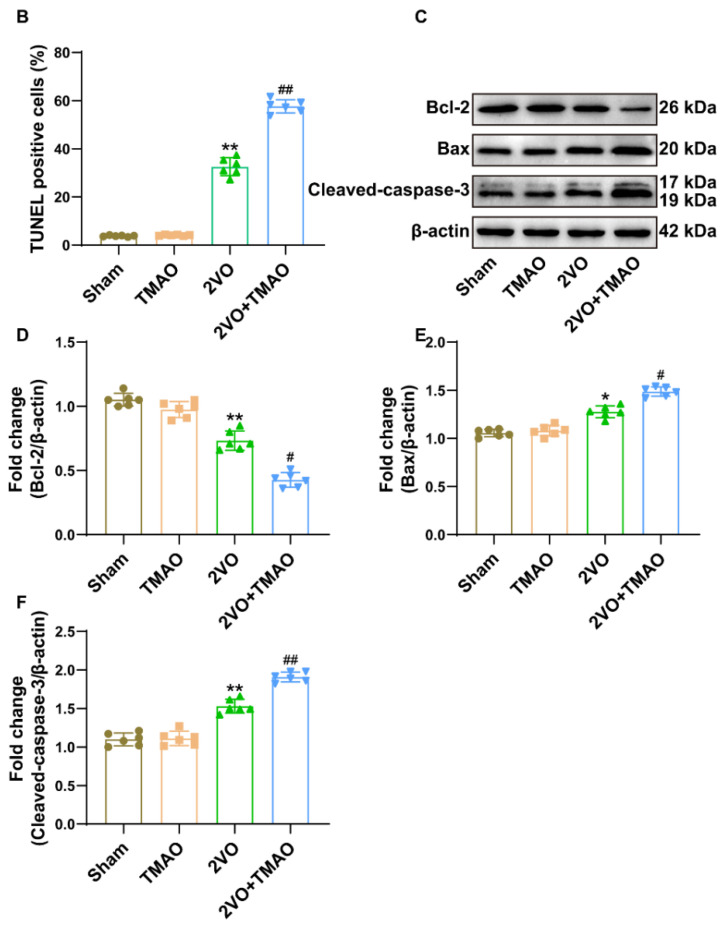
TMAO induced neuronal apoptosis within 2VO rats. (**A**) TUNEL staining images of the hippocampal CA1 area. Scale bar, 20 μm (n = 6). (**B**) Quantification of TUNEL-positive cells is shown as a bar chart (n = 6). (**C**) Representative bands of Bcl-2, Bax, and cleaved caspase-3 protein expression within the hippocampi of the rats across the groups. β-actin served as a loading control (n = 6). Quantification for (**D**) Bcl-2, (**E**) Bax, and (**F**) cleaved caspase-3 protein expression is shown as a bar chart (n = 6). Datasets reflect the mean ± SD. * *p* < 0.05 and ** *p* < 0.01 versus the Sham group; ^#^ *p* < 0.05 and ^##^ *p* < 0.01 versus the 2VO group.

We then assessed TMAO’s influence on neuronal apoptosis after 2VO. The dataset outcomes for TUNEL/NeuN staining showed that TMAO treatment could aggravate neuronal apoptosis within the CA1 area of rat hippocampi after 2VO surgery (Figure 4A,B). This investigation also probed apoptotic protein expressions within the hippocampal tissue of the 2VO rats. The data present upregulated Bax and cleaved caspase-3 expression within the 2VO group, together with downregulated Bcl-2. TMAO exposure further drove Bax and cleaved caspase-3 upregulation together with exacerbated Bcl-2 downregulation (Figure 4C,F). Such dataset outcomes suggest that TMAO disrupts the dynamic equilibrium of apoptotic genes, thereby promoting 2VO-induced apoptosis.

### 3.5. TMAO Promoted Hippocampal Oxidative Stress within 2VO Rats

**Figure 5:**
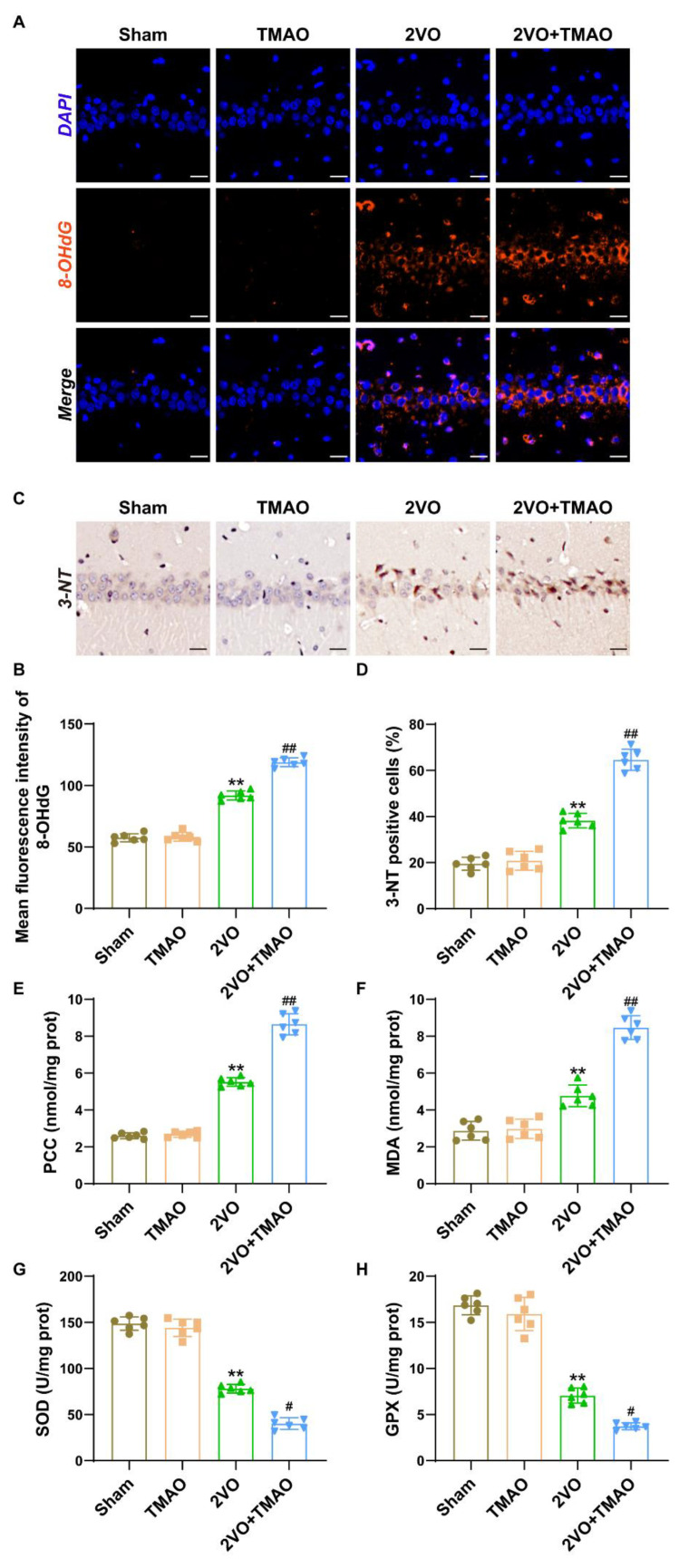
TMAO promoted hippocampal oxidative stress within 2VO rats. (**A**) Representative immunostaining of 8-OHdG (red) within the hippocampal CA1 area. Nuclei were subjected to DAPI staining. Scale bar, 20 μm (n = 6). (**B**) Quantification of the mean fluorescence intensity of 8-OHdG is shown as a bar chart (n = 6). (**C**) Imaging of the immunohistochemical staining demonstrates 3-NT expression within the hippocampal CA1 area. Scale bar, 20 μm (n = 6). (**D**) Quantification for 3-NT-positive cells is shown as a bar chart (n = 6). (**E**) PCC and (**F**) MDA levels, and (**G**) SOD and GPX (**H**) activity within the hippocampi of the rats were measured based on the kit manufacturer’s instructions (n = 6). Datasets reflect the mean ± SD. ** *p* < 0.01 versus the Sham group; ^#^ *p* < 0.05 and ^##^ *p* < 0.01 versus the 2VO group.

Oxidative stress injury occurs following the VaD model, so we tested whether there was a role for TMAO in 2VO-induced neuronal oxidative stress. Figure 5A–D depicts that 8-OHdG fluorescent expression and 3-NT-positive cells within the CA1 area of rats were markedly increased after 2VO, while the increments were largely promoted by TMAO treatment. Next, we examined the PCC and MDA levels, and SOD and GPX activity, in the hippocampal tissues after treatment with TMAO. The levels of PCC and the typical oxidative mediator MDA were clearly elevated within the 2VO group (Figure 5E,F). Conversely, the activity of SOD and GPX, two typical antioxidant enzymes, was notably suppressed within the 2VO group (Figure 5G,H). Treatment with TMAO further suppressed SOD and GPX activity and strikingly increased the PCC and MDA levels. These results suggest that TMAO could aggravate the oxidative stress injury of neurons within 2VO rat brains.

### 3.6. TMAO Drove Hippocampal NLRP3 Inflammasome Activation within 2VO Rats

**Figure 6:**
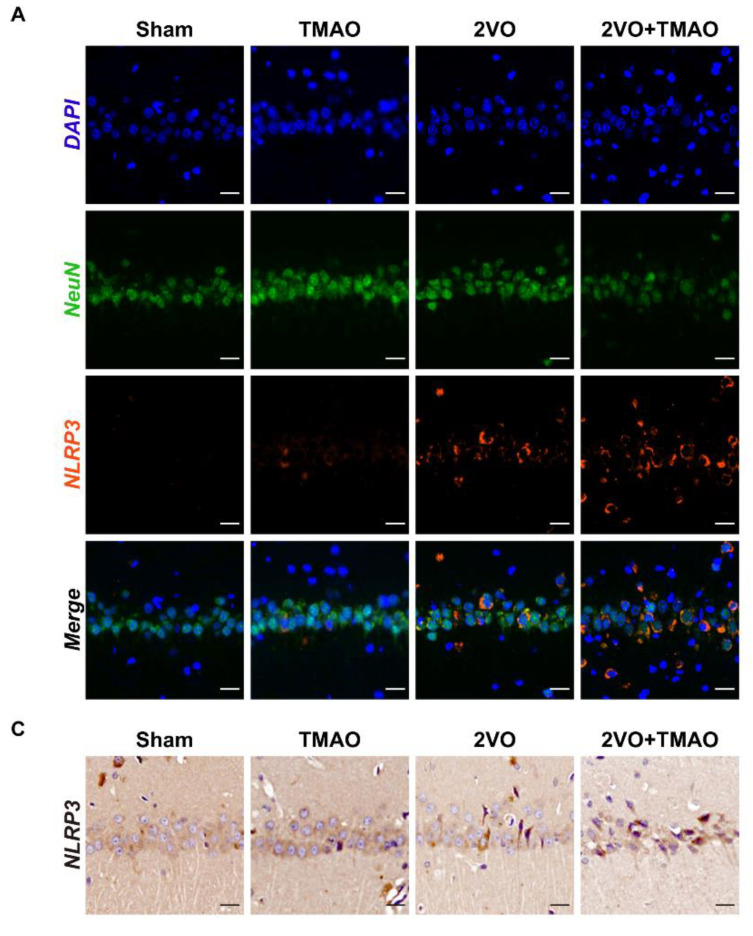

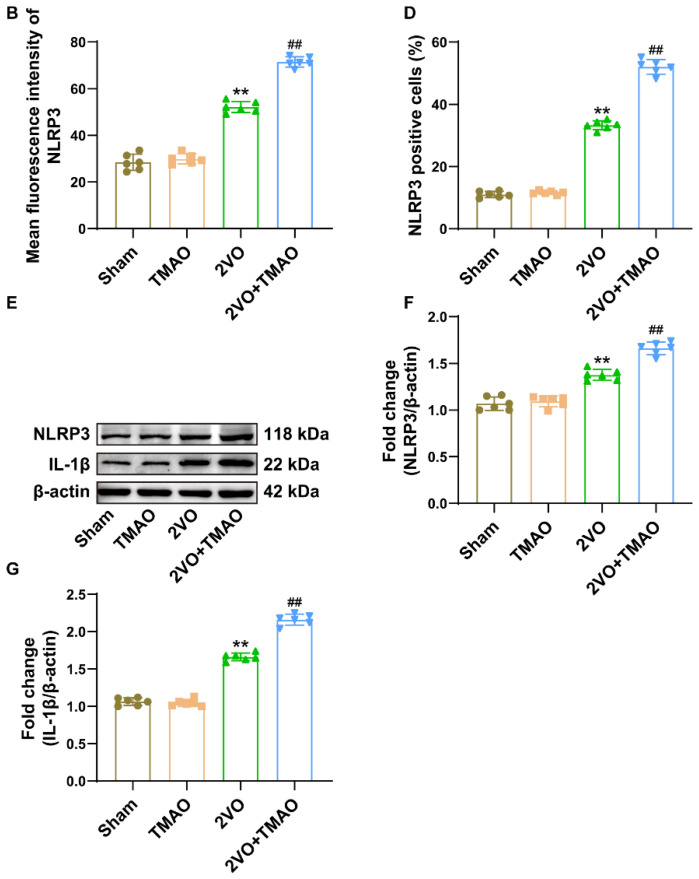
TMAO drove hippocampal NLRP3 inflammasome activation within 2VO rats. (**A**) Immunostaining displayed colocalization for NeuN (green) and NLRP3 (red) within the hippocampal CA1 area. Nuclei were subjected to DAPI staining. Scale bar, 20 μm (n = 6). (**B**) Quantification for the mean fluorescence intensity of NLRP3 is shown as a bar chart (n = 6). (**C**) Imaging of the immunohistochemical staining showing the NLRP3 expression within the hippocampal CA1 area. Scale bar, 20 μm (n = 6). (**D**) Quantification for the NLRP3-positive cells is shown as a bar chart (n = 6). (**E**) Representative bands of NLRP3 and IL-1β protein expressions within the rat hippocampi across the groups. β-actin served as a loading control (n = 6). Quantification for (**F**) NLRP3 and (**G**) IL-1β protein expression is shown as a bar chart (n = 6). Datasets reflect the mean ± SD. ** *p* < 0.01 versus the Sham group; ^##^ *p* < 0.01 versus the 2VO group.

Previous findings revealed that TMAO could trigger the NLRP3 inflammasome [20,34]. Regarding probing TMAO’s influence on NLRP3 expression within 2VO rat hippocampi, immunofluorescent assays, immunohistochemistry, and Western blots were performed to assess NLRP3 expression. Double immunofluorescence staining revealed that TMAO treatment could increase the NLRP3 expression in the neurons when compared to the 2VO group (Figure 6A,B). Likewise, immunohistochemistry confirmed the immunofluorescence results, showing that TMAO administration evidently enhanced the immunoreactivity of NLRP3 within 2VO rat hippocampi (Figure 6C,D). Furthermore, we measured the protein abundance of NLRP3 and IL-1β by Western blot. The protein expression of NLRP3 and IL-1β was noticeably upregulated within the 2VO rat hippocampi, which suggests that NLRP3 was activated after 2VO surgery (Figure 6E–G). As foreseen, the expression of these proteins was further upregulated after TMAO treatment. Such dataset outcomes provide further evidence for NLRP3 inflammasome signaling activation after 2VO, with TMAO promoting NLRP3 inflammasome activation.

### 3.7. TMAO Decreased Hippocampal SIRT1 Expression within 2VO Rats

**Figure 7:**
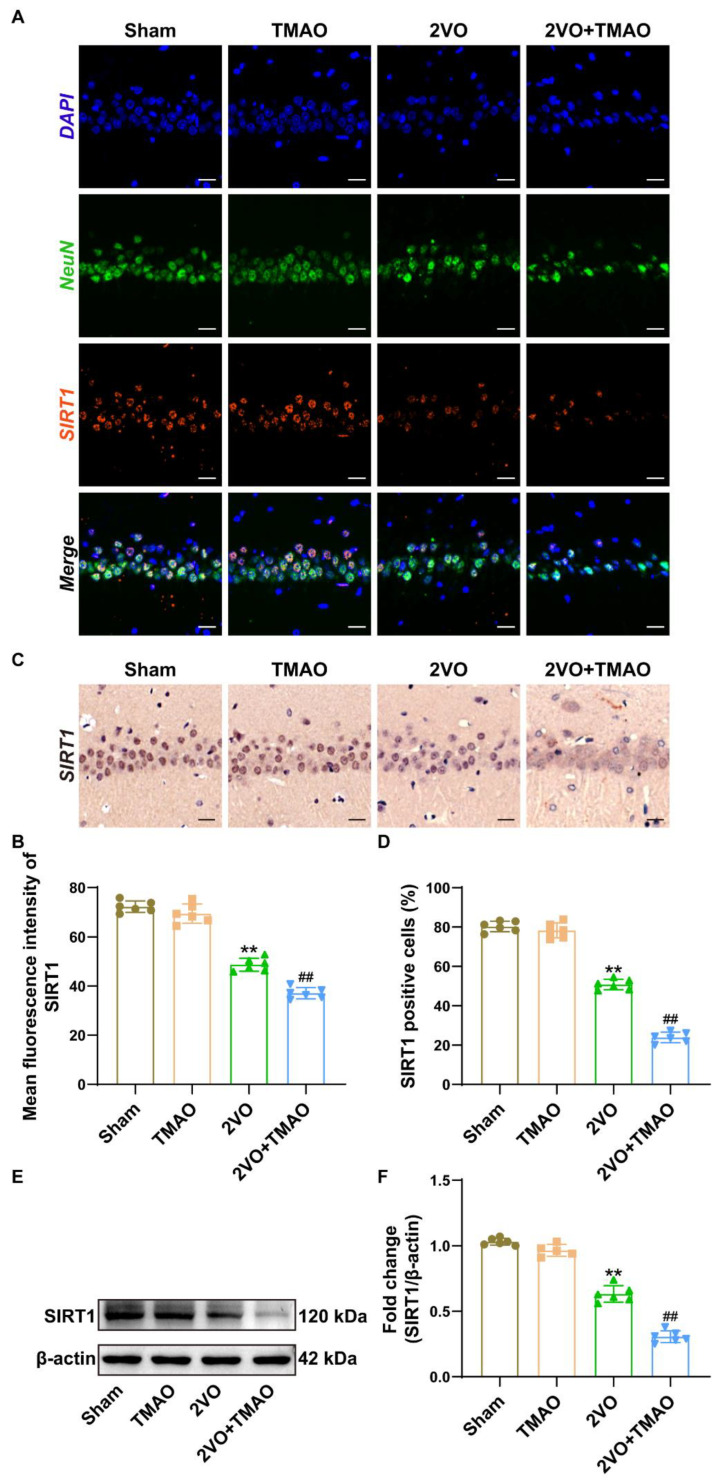
TMAO decreased hippocampal SIRT1 expression within 2VO rats. (**A**) Immunostaining displayed colocalization for NeuN (green) and SIRT1 (red) within the hippocampal CA1 area. Nuclei were subjected to DAPI staining. Scale bar, 20 μm (n = 6). (**B**) Quantification for the mean fluorescence intensity of SIRT1 is shown as a bar chart (n = 6). (**C**) Imaging of the immunohistochemical staining showing SIRT1 expression within the hippocampal CA1 area. Scale bar, 20 μm (n = 6). (**D**) Quantification for the SIRT1-positive cells is shown as a bar chart (n = 6). (**E**) Representative bands for SIRT1 protein expression within the rat hippocampi across the groups. β-actin served as a loading control (n = 6). (**F**) Quantification for SIRT1 protein expression is shown as a bar chart (n = 6). Datasets reflect the mean ± SD. ** *p* < 0.01 versus the Sham group; ^##^ *p* < 0.01 versus the 2VO group.

Regarding probing SIRT1 involvement in TMAO injury within 2VO rats, this study then investigated the changes in SIRT1 protein expression. The double immunofluorescence staining showed that TMAO treatment could further downregulate SIRT1 expression within hippocampal neurons in comparison to the 2VO group (Figure 7A,B). Meanwhile, the immunohistochemical staining of the rat brain tissue sections showed a similar trend, indicating that TMAO administration remarkably decreased SIRT1-positive cells within the 2VO group (Figure 7C,D). Furthermore, the Western blot outcomes displayed that the protein abundance of SIRT1 within the hippocampus of the 2VO rats was notably minimized, and TMAO treatment further reduced this expression level (Figure 7E,F). These outcomes imply that TMAO could downregulate SIRT1 expression within 2VO rat hippocampi.

### 3.8. TMAO Reduced Synaptic Plasticity and Promoted Neuronal Apoptosis by Inhibiting SIRT1 within 2VO Rats

**Figure 8:**
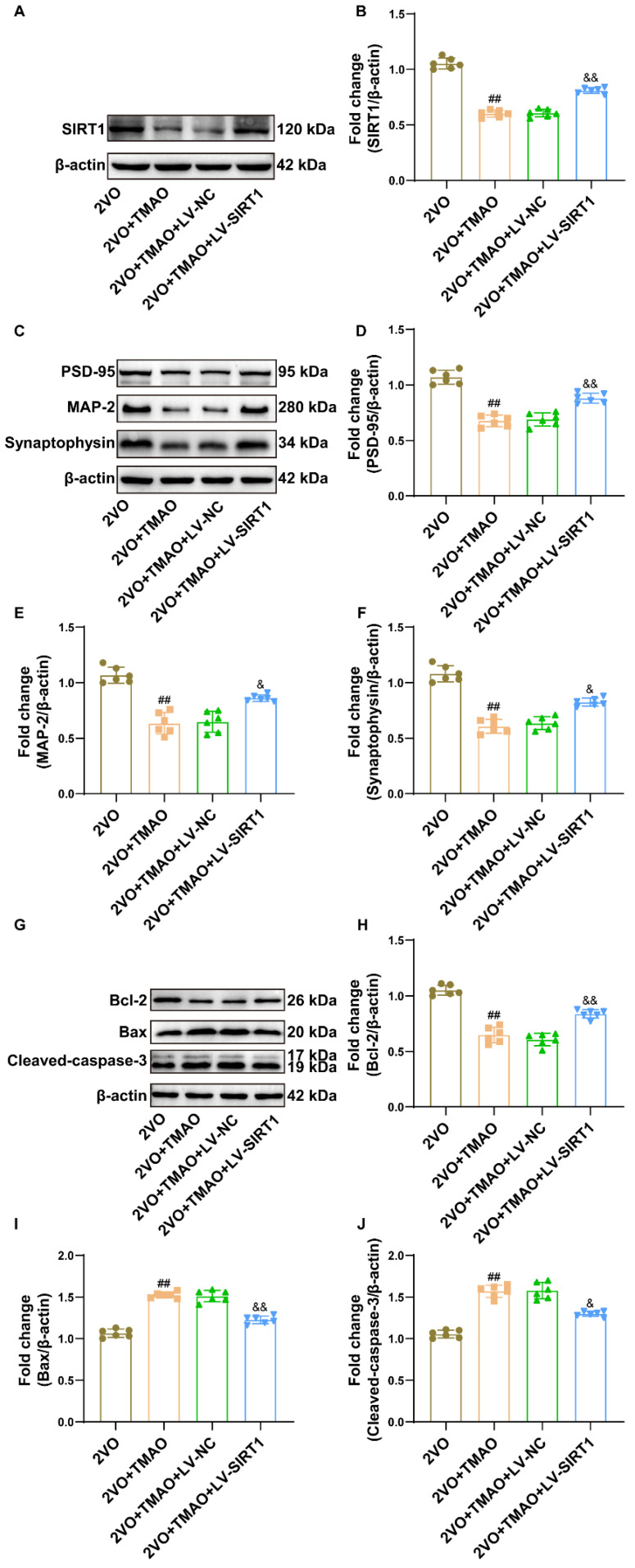
TMAO reduced synaptic plasticity and promoted neuronal apoptosis by inhibiting SIRT1 within 2VO rats. (**A**) Representative bands for SIRT1 protein expression within the rat hippocampi across the groups (n = 6). (**B**) Quantification for SIRT1 protein expression is shown as a bar chart (n = 6). (**C**) Representative bands of PSD95, MAP-2, and synaptophysin protein expression within the rat hippocampi across the groups (n = 6). Quantification for (**D**) PSD95, (**E**) MAP-2, and (**F**) synaptophysin protein expression is shown as a bar chart. β-actin served as a loading control (n = 6). (**G**) Representative bands for Bcl-2, Bax, and cleaved caspase-3 protein expression within the rat hippocampi across the groups. β-actin served as a loading control (n = 6). Quantification for (**H**) Bcl-2, (**I**) Bax, and (**J**) cleaved caspase-3 protein expression is shown as a bar chart (n = 6). Datasets reflect the mean ± SD. ^##^ *p* < 0.01 versus the 2VO group; ^&^ *p* < 0.05 and ^&&^ *p* < 0.01 versus the 2VO+TMAO+LV-NC group.

To demonstrate whether the above-mentioned effects of TMAO on the 2VO rats were mediated by SIRT1, SIRT1-overexpressing lentivirus (LV-SIRT1) was injected into the rat brains through the lateral ventricle. As indicated in Figure 8A,B, LV-SIRT1 dramatically upregulated the decrease in SIRT1 protein levels caused by TMAO. Subsequently, the Western blot outcomes display that TMAO treatment greatly reduced protein expression for PSD95, MAP-2, and synaptophysin, and that the overexpression of SIRT1 prevented those decreases (Figure 8C–F). Furthermore, LV-SIRT1 reversed TMAO-mediated aggravation of neuronal apoptosis (Figure 8G–J). These data suggest that TMAO reduces synaptic plasticity and promotes neuronal apoptosis in VaD rats by inhibiting SIRT1.

### 3.9. TMAO Promoted Neuronal Oxidative Stress and NLRP3 Inflammasome Signaling through a SIRT1-Based Pathway within 2VO Rats

**Figure 9:**
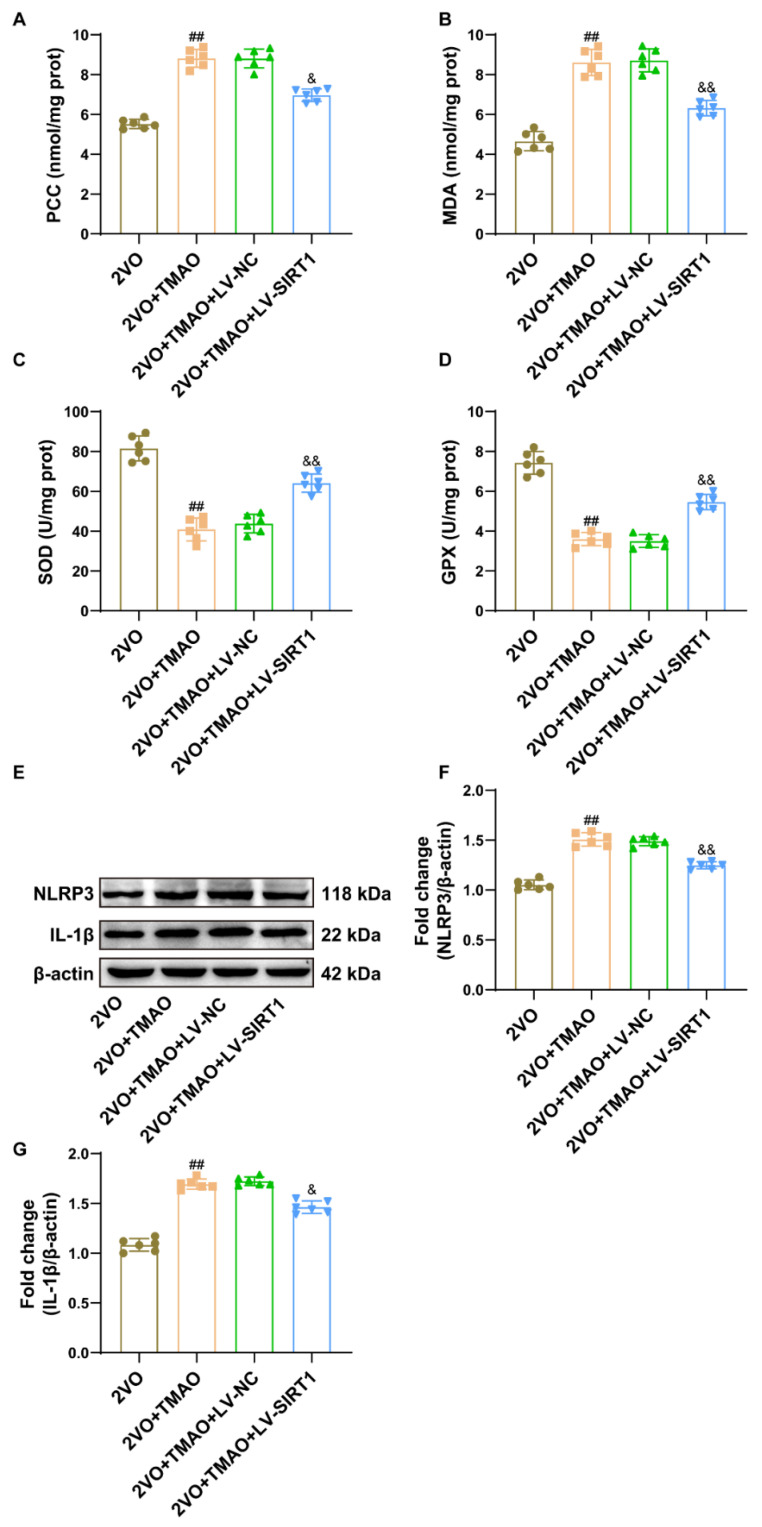
TMAO promoted neuronal oxidative stress and NLRP3 inflammasome signaling through a SIRT1-based pathway within 2VO rats. (**A**) PCC and (**B**) MDA levels, and (**C**) SOD and (**D**) GPX activity within the rat hippocampi were measured based on the kit manufacturer’s instructions (n = 6). (**E**) Representative bands of the NLRP3 and IL-1β protein expression within the rat hippocampi across the groups (n = 6). Quantification for (**F**) NLRP3 and (**G**) IL-1β protein expression is shown as a bar chart. β-actin served as a loading control (n = 6). Datasets reflect the mean ± SD. ^##^ *p* < 0.01 versus the 2VO group; ^&^ *p* < 0.05 and ^&&^ *p* < 0.01 versus the 2VO+TMAO+LV-NC group.

Next, we also found that the overexpression of SIRT1 abated the pro-oxidative effects of TMAO, as evidenced by increased SOD and GPX activity, and decreased levels of PCC and MDA (Figure 9A–D). We also examined the protein expression of the NLRP3 inflammasome by Western blot. The protein expression of NLRP3 and IL-1β was significantly upregulated after TMAO treatment, while this increase was clearly revoked by LV-SIRT1 (Figure 9E–G). This suggests that TMAO may promote neuronal oxidative stress damage and NLRP3 inflammasome signaling through a SIRT1-based pathway.

## 4. Discussion

This investigation confirmed that elevated TMAO in circulation could aggravate cognitive dysfunction in VaD rats. Cognitive dysfunction and nerve death were observed in postoperative rats following 2VO. When given TMAO for up to 8 weeks, we found that elevated circulating TMAO could impair both learning and memory functions and synaptic plasticity, together with enhancing 2VO-driven oxidative stress, inflammation, and apoptosis in VaD rats. Moreover, TMAO inhibited SIRT1 and upregulated the expression of NLRP3-regulated inflammation-related proteins, which may be the key mechanisms of TMAO nerve injury.

VaD is the second-most prevalent form of dementia stemming from long-term cerebral hypoperfusion [35]. The 2VO model can induce ischemia and hypoxia injury in brain tissues such as the hippocampus and cortex and has been widely used in VaD research [36]. As a metabolite of gut microbes, the role of TMAO in central nervous system diseases has been receiving more attention [37]. One investigation revealed that TMAO upregulation within AD patients was positively linked to AD pathology and neurodegenerative biomarkers [19]. In addition, DMB, a TMAO inhibitor, could diminish circulating TMAO levels to attenuate the memory cognitive functional decline within APP/PS1 mice [38]. However, the role of pre-existing, higher, circulating TMAO in VaD is not known. In this paper, we observed that TMAO treatment remarkably increased the plasma TMAO at 4 weeks after surgery, which was also confirmed within the 2VO rat hippocampi. Simultaneously, the OFT and MWM results illustrated that TMAO treatment further aggravated cognitive dysfunction in the VaD rats. Synaptic plasticity reduction and synaptic plasticity-associated decreases in protein synthesis are major contributors to cognitive dysfunction within the brain. Notably, Li et al. revealed that elevated TMAO levels showed mechanistic relevance to cognitive dysfunction by aggravating synaptic damage [22]. Through Golgi staining, we observed that elevated circulating TMAO led to apical dendritic spine loss in the 2VO rats’ hippocampal CA1 area. Moreover, we determined the protein expression for synaptic plasticity-associated proteins. Similarly, our Western blot data suggested that elevated circulating TMAO could downregulate synaptic plasticity-associated proteins, including synaptophysin, MAP-2, and PSD-95, which led to cognitive deficits in the rats.

Growing evidence has suggested that oxidative stress is a crucial pathological factor in VaD, which often causes highly severe impairment to neurons [39,40]. Aggregations of numerous reactive oxygen species (ROS) lead to the abnormal circulation of NO and aggravate the damage of VaD [41]. Previous studies have reported that circulating TMAO could traverse the blood–brain barrier for driving oxidative stress and consequent cerebral injury [22,42]. For example, TMAO could exacerbate oxidative stress through downregulating methionine sulfoxide reductase A (MsrA) within the hippocampus [43,44]. In line with past investigations, this study identified upregulated 8-OHdG, 3-NT, PCC, and MDA expression, while SOD and GPX activity was reduced in the VaD rats. The TMAO treatment further aggravated these situations. Moreover, oxidative mediators act as messengers that drive inflammasome activation and have a pivotal role in NLRP3 inflammasome activation [45]. Triggered NLRP3 inflammasome converts pro-caspase-1 into functional caspase-1, thereby promoting IL-1β maturation, as well as triggering inflammatory and immune responses [46,47,48]. Meanwhile, a large amount of rapidly generated oxidative substances can activate the NLRP3 pathway to further induce apoptosis [49]. Interestingly, TMAO could drive oxidative stress to trigger NLRP3 inflammasome signaling, leading to pro-inflammatory cytokine discharge [21]. In addition, previous studies uncovered that TMAO could reduce cell viability and promote apoptosis within MPC-83 cells [50], and TMAO was reported to aggravate hyperoxaluria-induced renal impairment via increasing, oxidative, stress-induced apoptosis [51]. However, whether the effects of elevated circulating TMAO on vascular dementia involve inflammasome activation and apoptosis remains to be elucidated. Aiming to explore the effect of elevated circulating TMAO on the NLRP3 inflammasome and apoptosis of VaD rats, this investigation probed for the expression of relevant proteomic biomarkers and TUNEL-positive cells. Our findings indicated that the 2VO model triggered NLRP3 inflammasome activation and apoptosis, which was consistent with previous findings [52]. In contrast, TMAO administration seemed to further enhance inflammasome activation and apoptosis.

SIRT1 is an NAD^+^-dependent class III histone deacetylase with a critical regulatory role within the CNS and cerebral functional development. Currently, accumulating data suggest that SIRT1 is involved in neurogenesis and synaptic plasticity [53,54]. Moreover, as a multifunctional protein, it participates in important physiological functions such as metabolism, aging, oxidative stress, apoptosis, and inflammation through the deacetylation of histones and non-histones [55]. Indeed, the neurovascular protective benefit of SIRT1 has already been linked to a cerebral perfusion deficit due to bilateral common carotid artery stenosis [56]. Additionally, Ke and colleagues observed that elevated circulating TMAO levels may exacerbate endothelial cell senescence along with vascular aging through the inhibition of SIRT1 expression and increase in oxidative stress [23]. However, whether SIRT1 is implicated in the destructive action of TMAO on 2VO rats remains to be identified. The outcomes of the data from this study clearly displayed that the 2VO model markedly downregulated SIRT1 within the hippocampus, whereas this inhibition was enhanced after TMAO treatment. In addition, the promotional effects of TMAO on 2VO-induced synaptic damage, oxidative stress, NLRP3 inflammasome activation, and apoptosis were abolished when the SIRT1 pathway was overexpressed through the intracerebroventricular injection of the LV-SIRT1 lentivirus. Such dataset outcomes indicate that the damaging influence of elevated circulating TMAO on VaD rats is SIRT1-dependent.

It should be noted that this study has some limitations, including that oxidative stress can stimulate neurogenesis in the initial stage, but we were more concerned about apoptosis in the study. Therefore, neurogenesis will be investigated in future research.

In conclusion, our findings uncovered that TMAO deteriorated 2VO-induced cognitive dysfunction through the disruption of synaptic plasticity, increasing oxidative stress and apoptosis, together with promoting the activation of the NLRP3 inflammasome. Importantly, our findings show that the deleterious effects of TMAO on 2VO rats were mediated by SIRT1. Collectively, the prevalence of elevated levels of circulating TMAO impaired cognitive function in vascular dementia rats through the downregulation of SIRT1.

## Data Availability

All data generated or analyzed during this study are available from the corresponding author upon reasonable request.
